# Genetic Diversity and Fine-Scale Genetic Structure of *Spodoptera litura* Fabricius (Lepidoptera: Noctuidae) in Southern China Based on Microsatellite Markers

**DOI:** 10.3390/ani13040560

**Published:** 2023-02-05

**Authors:** Zhongwen Hu, Fangyuan Yang, Deping Zhang, Shimeng Zhang, Xiaofei Yu, Maofa Yang

**Affiliations:** 1 Guizhou Provincial Key Laboratory for Agricultural Pest Management of Mountainous Region, Institute of Entomology, Scientific Observing and Experimental Station of Crop Pest in Guiyang, Ministry of Agriculture and Rural Affairs, Guizhou University, Guiyang 550025, China; 2 Science and Technology Department, China National Tobacco Corporation Guangxi Zhuang Autonomous Region Company, Nanning 530022, China; 3 College of Tobacco Science, Guizhou University, Guiyang 550025, China

**Keywords:** *Spodoptera litura*, microsatellite marker, genetic differentiation, gene flow

## Abstract

**Simple Summary:**

*Spodoptera litura* is an important agricultural pest widely distributed around the world, which can damage numerous crops. Despite its economic importance, research on the population genetics of *S. litura* has been limited, preventing insight into the spread of the pest. In this study, we conducted a population genetic analysis of 24 populations of *S. litura* in Southern China using microsatellite markers, demonstrating the information on the genetic diversity and genetic structure of the tested populations. The results will provide pest control management strategies for *S. litura* in Southern China.

**Abstract:**

Population genetic structure is strongly affected by dispersal events, especially for migratory species. The investigation of population structure is therefore conducive to increasing our understanding of species dispersal. *Spodoptera litura* (Fabricius) (Lepidoptera: Noctuidae) is an important tobacco pest in China causing serious damage to multiple crops. In this study, we explore its dispersal dynamics by clarifying the fine-scale population genetics using 545 *S. litura* samples collected from tobacco plantations at 24 locations (mainly in Baise, Hechi, and Hezhou, Southern China). We analyzed the genetic diversity, genetic structure, and gene flow of these populations using seven microsatellite loci. Our results revealed high genetic diversity and low population genetic structure among *S. litura*. The genetic distance was uncorrelated with geographical distance, indicating the complete randomness of dispersal among the local populations. Our results suggest that the movement scope of contemporary *S. litura* might be much higher than the local-level spatial scale, which will provide a theoretical basis for pest management.

## 1. Introduction

Population genetics is the study of changes in gene frequency and genotype frequency in a biological population under the influence of natural selection, genetic drift, mutation, and migration [1]. Population genetic structure is the main research subject of population genetics, which refers to the distribution pattern of genetic variations among and within populations [2]. Many insects exhibit unique dispersal patterns. Some, like *Grapholita molesta* [3] and *Cydia pomonella* [4], are weak flying insects that can only move in a small range, whereas other insects such as *Mythimna seperata* [5], *Leptinotarsa decemlineata* [6], and *Spodoptera exigua* [7] with extremely strong flying ability, are not affected by external environmental pressures and are often able to migrate long distances. However, the long-distance dispersal of insects promotes gene flow, evolutionary potential, and the spread of resistance genes at a regional and large spatial scale. This not only increases the difficulty of management strategies but also harms agricultural economics [8,9]. Population genetic structure is a powerful means of obtaining information on the long-distance movement of insects and helps to understand their dispersal patterns and capabilities over geographic patterns to provide effective prevention and control measures.

Molecular marker techniques are widely used in population genetics as an important means for studying genetic variations and the degree of genetic differentiation among populations [10]. Compared to other molecular marker technologies, microsatellites with the characteristics of good specificity, high polymorphism, and co-dominant inheritance, are often used to analyze population genetic variations, genetic structure, and migration patterns [11,12]. For example, Jiang et al. used microsatellite technology to analyze the genetic variation and population genetic structure of *Laodelphax striatellus* in 15 regions of northeastern China and observed a certain degree of genetic differentiation and variation in genetic structure among populations [13].

The tobacco cutworm, *Spodoptera litura* (Fabricius) (Lepidoptera: Noctuidae), is an omnivorous and gluttonous agricultural pest, mainly distributed in the Middle East, most of Asia, America, Africa, and the South Pacific [14,15,16,17]. In China, *S. litura* was first recorded in Hubei province in 1900 [18]. At present, it has been colonized in all provinces of China, except Tibet. The larvae of *S. litura* feed on leaves, flowers, buds, and fruits and can attack 389 species of host plants from 109 families, including tobacco, pepper, taro, peanuts, and other cash crops [19,20]. *Spodoptera litura* is a crucial pest in major tobacco-growing regions of Southern China. Large outbreaks, consequently related to high reproductive ability and long-distance migration, lead to serious economic losses to local growers [20]. The current knowledge on *S. litura* involves various aspects of its occurrence and damage, biology, ecology, and control techniques, but knowledge of the population genetics of *S. litura* is limited, which hinders the in-depth understanding of pest dispersal. Moreover, previous studies on the population genetics of *S. litura* have been conducted mainly at large spatial scales [21,22], and few studies have been reported on local and regional spatial scales.

In this present study, seven microsatellite loci were used to elucidate population genetic differentiation using fine-scale data modeling in Southern China. We analyzed the genetic diversity, genetic structure, and gene flow of the population. These studies will help to clarify the occurrence dynamics of *S. litura* in local areas, providing a theoretical basis for the dispersal blocking and control of the pest.

## 2. Materials and Methods

### 2.1. Sample Collection and DNA Extraction

**Figure 1:**
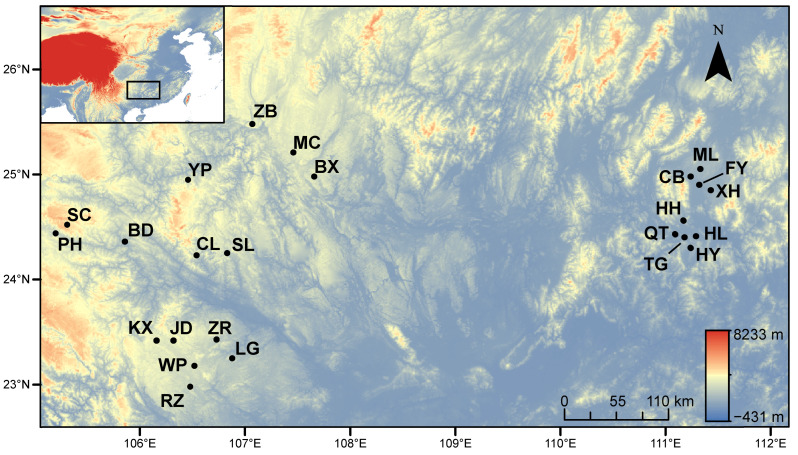
Geographical locations of 24 sampled populations of *S. litura*. The codes of towns represent different populations. Hezhou city: Mailing (ML), Xinhua (XH), Fuyang (FY), Chengbei (CB), Honghua (HH), Qingtang (QT), Tonggu (TG), Huilong (HL), Huangyao (HY); Hechi city: Baxu (BX), Mangchang (MC), Zhongbao (ZB); Baise city: Badu (BD), Youping (YP), Puhe (PH), Chaoli (CL), Shali (SL), Shechang (SC), Jingde (JD), Zurong (ZR), Longguang (LG), Renzhuang (RZ), Wuping (WP), Kuixu (KX).

A total of 545 *S. litura* larvae were collected from tobacco plants in 24 townships of Baise City, Hezhou City, and Hechi City in Guangxi Zhuang Autonomous Region, Southern China (Figure 1 and Table 1). We collected 14–30 individuals for each population, with an interval of more than 3 m between each individual to avoid collecting the same clone. Genomic DNA was extracted from the head of each larva using Ezup Column Animal Genomic DNA Purification Kit (Shanghai, China) according to the manufacturer’s instructions.

### 2.2. PCR Amplification and Genotyping

The nine pairs of microsatellite primers [23], seven efficient pairs (CWM−1, CWM−4, CWM−5, CWM−6, CWM−7, CWM−8, and CWM−9) were selected for PCR amplification and downstream analysis. Each forward primer was labeled with a fluorescent dye (5′−FAM, NED, ROX, or HEX). PCR reactions were performed in a 25 μL volume containing 13 μL 2 × Taq PCR Master Mix, 1 μL DNA template, 1 μL forward and reverse primers, and 9 μL ddH_2_O. The cycling conditions were executed with a pre-degeneration at 95 °C for 4 min, denaturation of 95 °C for 30 s, followed by 30 cycles of 30 s at 94 °C, 30 s at the appropriate annealing temperature, ending with elongation at 72 °C for 7 min. The PCR products were sequenced using ABI3730XL automatic sequencer by Sangon Biotech (Shanghai, China). GeneMarker 2.4 [24] was used to manually read and check the lengths of microsatellite fragments.

### 2.3. Statistical Analyses

#### 2.3.1. Genetic Diversity

Micro−checker [25] was used to evaluate null alleles and correct genotypic errors. Deviation from Hardy–Weinberg equilibrium (HWE) [26] at each locus in populations was calculated using the program GenAlEx 6.5 [27]. Linkage disequilibrium (LD) between pairs of loci was performed in GENEPOP 4.6 [28]. The number of alleles (*N*_a_), polymorphism information content (PIC), and Shannon’s index (*I*) were estimated using Microsatellite Tools (Trinity College Dublin, Ireland) and GenAlEx. Observed heterogeneity (*H*_O_), expected heterozygosity (*H*_E_), and allelic richness (*A*_R_) were assessed using the *hierfstat* R package [29].

#### 2.3.2. Genetic Structure and Population Differentiation

STRUCTURE 2.3.4 [30] 30 was used to assess the genetic structure of populations based on the Bayesian model. The candidate number of genetic clusters (*K*) was set as 1 to 10 with 10 repeat runs and a burn-in period of 100,000 and 1,000,000 Monte Carlo Markov Chains. The results were submitted to Structure Harvester Web 0.6.93 [31] to confirm the optimal K value by the ∆*K* method [32]. The membership coefficient matrices (Qmatrices) of repeated runs associated with the optimal *K* were integrated using CLUMPP 1.12 [33], and the results were visualized using DISTRUCT 1.1 [34]. In addition, a Principal Coordinates Analysis (PCoA) was performed based on Codom−Genotypic genetic distance among 545 *S. litura* populations using GenAlEx.

The analysis of molecular variance (AMOVA) was calculated by ARLEQUIN 3.5 [35] to assess the distribution of genetic variance between populations, individuals, and groups. The F-statistics (*F*_st_) and the Nei’s genetic distance (*D*’) per geographical population were estimated using Fstat [36] and Popgene32 [37], respectively.

The geographical distance among different populations was calculated based on latitude and longitude. A Mantel test, based on geographic distances and linearized pairwise *F*_ST_ (i.e., *F*_ST_/1 − *F*_ST_) [38], was conducted to explore the effect of isolation by distance (IBD). To test the effect of IBD in different spatial ranges, we also performed a spatial autocorrelation analysis using the PopGenReport R package [39]. We divided geographic distances into several bins with a step distance of 45 km and independently tested the correlations with corresponding genetic distances in the subset bins.

#### 2.3.3. Gene Flow

BAYESASS 3.0 [40] with Markov chain Monte Carlo method was used to evaluate the modern migration rates among *S. litura* populations. The program was run with 10,000,000 iterations, discarding the first 1,000,000 iterations, and sampling every 1000 iterations from the remaining 9,000,000 iterations, producing a sample of 9000 observations from the chain that will be used to estimate parameters.

## 3. Results

### 3.1. Genetic Diversity of Populations

In all loci-population pairs, only 42 of 168 have significantly deviated from Hardy–Weinberg equilibrium, which was caused by heterozygote deficiency. Linkage disequilibrium occurred in 3 of 21 locus-locus pairs, including CWM−1 versus CWM−4, CWM−1 versus CWM−8, and CWM−7 versus CWM−9. However, no loci had significant global deviation linkage disequilibrium, so the independence of the loci was sufficient. The genetic diversity of 24 populations of *S. litura* is displayed in Table 2. The number of alleles (*N*_a_) ranged from 4.14 to 6.86 with an average of 5.73. The mean number of observed heterogeneity (*H*_O_) and expected heterozygosity (*H*_E_) were 0.4793 and 0.5970, respectively. The average of Shannon’s index (*I*) was 1.2342. The highest allele richness (*A*_R_) was 5.7783 in ZB population, while the lowest *A*_R_ was 4.1429 in SC population. Polymorphism information contents (PIC) of all populations were greater than 0.5.

### 3.2. Population Genetic Structure

**Figure 2:**
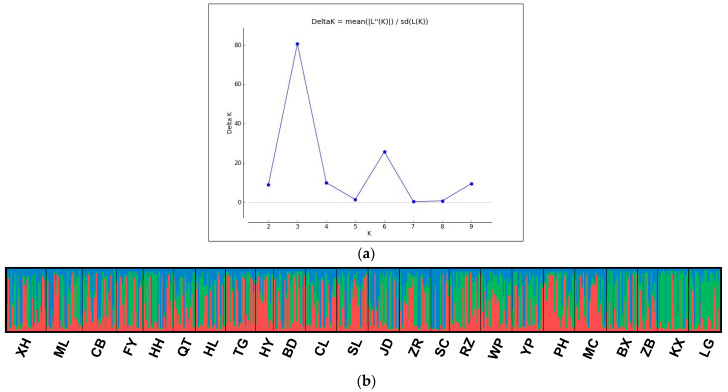
(**a**). Estimates of optimal *K* (number of clusters) from Δ*K*. (**b**). The structure bar shows the results of the Bayesian analysis of 24 populations of *S. litura.* Each individual is shown by a vertical bar. Blue, green and red represent the three clades.

**Figure 3:**
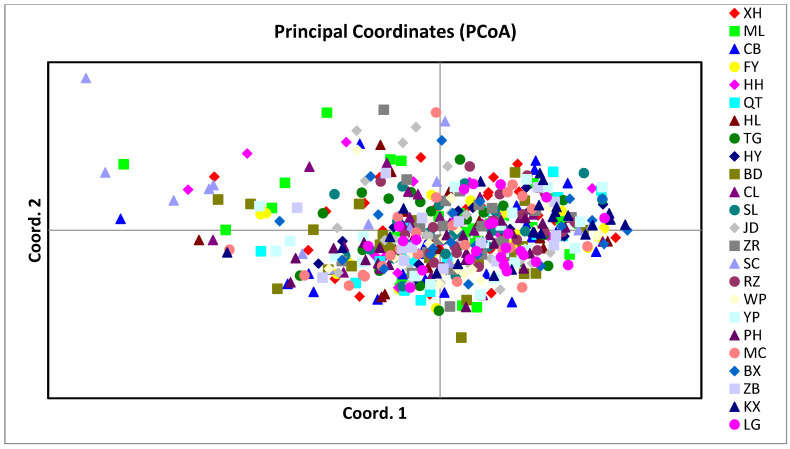
Individualbased principal coordinate analysis from 24 *S. litura* populations.

Based on the Bayesian cluster analysis using STRUCTURE software, the optimal genetic clusters K = 3 was detected (Figure 2a). However, there were no clear geographical grouping patterns for all populations (Figure 2b). Such mixed patterns also occur when K equals values other than 2–10. Similarly, the principal coordinate analysis (PCoA) also had undefined grouping patterns, showing that different geographical populations were mostly mixed. These results indicated that the clustering pattern was not related to sampling locations (Figure 3).

### 3.3. Population Differentiation

Based on the AMOVA, it showed a 1.92% of variation among populations, 20.02% of variation among individuals within populations, and 78.06% of variation among individuals (Table 3). These results suggest that genetic variation mainly occurred within individuals. Two genetic distances measured showed a low level of differentiation among all populations (Table 4). Pairwise *F*_ST_ values ranged from 0.013 to 0.130. *D*′ ranged from 0.019 to 0.238. In particular, the CB and BD populations showed the lowest differentiation (*F*_ST_ = 0.013, *D*′ = 0.019), and The SC and KX populations showed the highest differentiation (*F*_ST_ = 0.130, *D*′ = 0.238).

**Figure 4:**
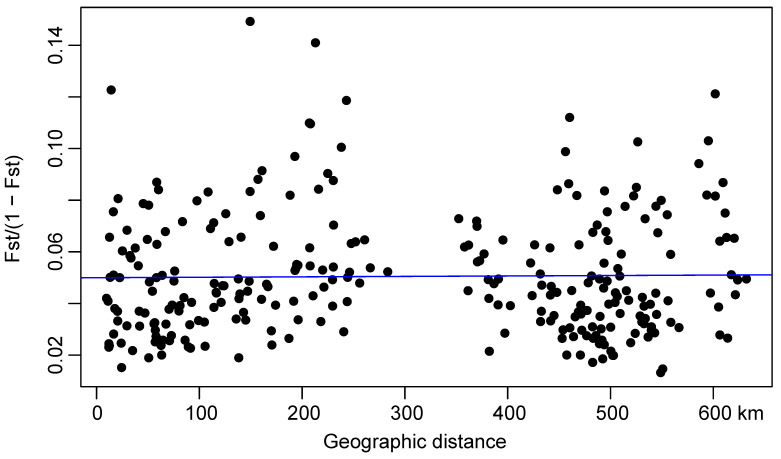
Mantel test between geographical distances and *F*_ST_/(1 − *F*_ST_). (r = 0.0145, *p* = 0.4).

The Mantel test of *F*_ST_/(1 − *F*_ST_) against natural log-transformed geographic distance (Ln) between populations revealed that genetic differentiation was weak-positively correlated with geographic distance (r = 0.0145, *p* = 0.4) (Figure 4). Spatial auto-correlation analysis showed a very weak positive correlation at 89 km and 622 km (Table 5).

### 3.4. Gene Flow

As shown (Table 6), we marked the migration rates of all populations by color shades. The results indicated that the range of recent migration rates for *S. litura* populations was 0.0061–0.1536. The self-assignment rate in the KX population was the highest (0.7868), and the two highest migration rates were calculated from LG to KX population. In addition, we found that most populations tend to migrate to Chengbei regions (CB).

## 4. Discussion

Owing to the neutral and high mutation rates of microsatellite markers, they are usually used to explore the level of genetic diversity and population differentiation within species [41,42]. In this study, the microsatellite molecular technique was used to explore 24 geographical populations of *S. litura* in Southern China, showing high genetic diversity and low genetic population structure.

### 4.1. Genetic Diversity

The higher the genetic diversity of the species, the stronger it’s potential to adapt to environmental changes [43,44]. In this study, the mean *H*_E_ for the *S. litura* population was 0.5970 and the average allelic richness (*A*_R_) of the *S. litura* populations was 5.1439, showing a high level of population genetic diversity. For other *S. litura* populations in China, genetic diversity was found to be generally high (*H*_E_ from 0.19 to 0.89) by Wu et al., which suggested that the rich genetic diversity was mainly due to frequent individual migration [23]. In addition, the excess of heterozygotes indicated that the strong ability of *S. litura* migration avoided severe bottlenecks and loss of rare alleles. We further suggested the importance of high-level gene flow in maintaining local population diversity, which may be one of the reasons for population outbreaks and insecticide resistance [45].

### 4.2. Genetic Structure and Population Differentiation

Based on the Bayesian clustering analysis, 24 populations in Southern China formed three clusters. However, there was no obvious geographic division pattern in the distribution of these three clusters, indicating that the geographic factors had little influence on the migration of *S. litura* in the study area. The PCoA results also provided similar evidence. Such patterns were observed in *Helicoverpa gelotopoeon* [46] and *Sitobion avenae* [47], where the population structure was unrelated to geographical distribution, which is speculated to be formed through extensive migration. Indeed, continued long-term dispersal can gradually homogenize populations, leading to a reduction in population structure [48]. This finding was inconsistent with Scott et al. [49] and Domingues et al. [50]. These results may be attributed to the long-distance dispersal and migration of insects leading to a low genetic divergence in population structure, forming a panmictic population.

Frequent migrations may offset the effects of genetic drift and mutation, thus reducing population genetic differentiation [51]. Our study showed that low genetic differentiation existed in the *S. litura* populations based on the local-level spatial scale. Among them, genetic differentiation did not exist in XH and PH populations, even though they were more than 600 km apart. Similarly, Gandhi and Patil [21] found high similarities between the Hyderabad and Indore populations of *S. litura* in India, which were about 670 km apart. Moreover, according to Wu [22], genetic undifferentiating was found among the 18 populations of *S. litura* in China, although the distance between the two farthest populations (Kunming and Shenyang populations) was about 2600 km. Therefore, we speculated that the genetic differentiation of *S. litura* may increase at a distance of more than 2600 km. How does our research on genetic differentiation measure up with other moths with high dispersal rates? Normally, moths with high dispersal rates exhibit lower *F_ST_*. We discovered that there was no genetic divergence between populations at similar or much larger geographic scales than we studied. For example, Franklin et al. [52] also observed that populations of *Trichoplusia ni* on the west coast of North America remained linked at distances greater than 600 km. However, some species began to diverge at scales greater than the national-level spatial scale. Lyons et al. [53] reported high genetic similarity between populations of the emperor butterfly in eastern and western North America, but significant genetic differentiation between them and populations in Hawaii and New Zealand, 4000 km away. According to Enderby et al. [48], it can be concluded that there was no significant genetic divergence between the Australian populations of *Plutella xylostella* with geographic distances of over 3600 km, however, significant divergence occurred in these populations with the Malaysia and Kenya populations, which were 5000 km away. Understanding the degree of genetic differentiation of such moths with high migratory capabilities at the geographic scale will help to precisely track the approximate extent of population expansion and provide a basis for future pest management planning.

### 4.3. Gene Flow

The extensive gene flow revealed by our analysis may confirm the cause of weak genetic differentiation. Migration rates between populations were highly correlated with pairwise *F*_ST_ [54], indicating the major role of gene flow in causing genetic differentiation. The high level of migration and broad environmental plasticity of *S. litura* populations that introduced genetic variations into partly local populations facilitated gene flow between populations. The high level of gene flow allowed resistance genes to disperse over long distances, leading to widespread dispersion of some chemical pesticide resistance genes, which may have resulted in adaptation to control strategies. The tobacco planting areas in Guangxi are mainly concentrated in Baise, Hechi, and Hezhou City with suitable geographical conditions for tobacco growth. These populations are mostly located in mountains, plateaus, and a few plains, among which Hechi and Baise Cities, near the Yunnan-Guizhou Plateau with rich Karst landscapes. Despite the complexity of geographical factors, the influence on the genetic differentiation of populations was minimal. We infer that the spread of these populations may be related to wind, climate, and human interference. It is noteworthy that high levels of gene flow can easily cause repeated outbreaks under appropriate conditions due to the transmission of rapidly resistant alleles among populations.

## 5. Conclusions

Our study showed high genetic diversity and low genetic differentiation of *S. litura* populations in Southern China. Overall, we consider that *S. litura* populations form a panmictic population on account of their long-distance migration, sufficient gene exchange, and low genetic differentiation. This helps explain the perennial susceptibility of *S. litura* to large outbreaks, resulting in extremely difficult prevention and control in local areas. Therefore, we suggest that, for migratory pests, the implementation & scope of management should be expanded to achieve the desired effect.

## Figures and Tables

**Table 1:** Sampling districts and the number of specimens of *S. litura* used in this study.

City	Population	Longitude	Latitude	Sample Size	Date
Hezhou	ML	111.33° E	25.05° N	28	2022.06
Hezhou	XH	111.43° E	24.85° N	30	2022.06
Hezhou	FY	111.32° E	24.90° N	20	2022.06
Hezhou	CB	111.24° E	24.98° N	26	2022.06
Hezhou	HH	111.17° E	24.56° N	23	2022.06
Hezhou	QT	111.09° E	24.43° N	17	2022.06
Hezhou	TG	111.18° E	24.40° N	23	2022.07
Hezhou	HL	111.29° E	24.41° N	23	2022.05
Hezhou	HY	111.24° E	24.30° N	14	2022.07
Hechi	BX	107.66° E	24.98° N	24	2022.06
Hechi	MC	107.46° E	25.21° N	24	2022.06
Hechi	ZB	107.07° E	25.48° N	15	2022.06
Baise	BD	105.86° E	24.36° N	24	2022.05
Baise	YP	106.46° E	24.95° N	24	2022.06
Baise	PH	105.20° E	24.44° N	24	2022.05
Baise	CL	106.54° E	24.23° N	24	2022.05
Baise	SL	106.83° E	24.25° N	24	2022.05
Baise	SC	105.31° E	24.52° N	14	2022.07
Baise	JD	106.32° E	23.42° N	24	2022.05
Baise	ZR	106.73° E	23.43° N	24	2022.05
Baise	LG	106.88° E	23.25° N	24	2022.05
Baise	RZ	106.48° E	22.98° N	24	2022.05
Baise	WP	106.52° E	23.18° N	24	2022.05
Baise	KX	106.16° E	23.42° N	24	2022.05

**Table 2:** Genetic diversity in the 24 populations of *S. litura*.

Population	*n*	*N*_A_	*H*_O_	*H*_E_	*A*_R_	PIC	*I*
XH	30	6.86	0.4690	0.6405	5.6761	0.6028	1.3729
ML	28	6.29	0.4261	0.6103	5.3120	0.5681	1.2786
CB	26	6.71	0.4176	0.6002	5.6280	0.5649	1.2999
FY	20	5.14	0.4820	0.5799	4.8323	0.5349	1.1677
HH	23	5.86	0.4848	0.6038	5.1305	0.5606	1.2457
QT	17	5.14	0.3613	0.5635	4.8244	0.5138	1.1130
HL	23	5.71	0.5590	0.6215	5.2608	0.5767	1.2789
TG	23	6.29	0.4596	0.5836	5.4250	0.5445	1.2334
HY	14	4.57	0.4184	0.5601	4.5714	0.5151	1.1226
BD	24	5.57	0.4501	0.5991	4.9598	0.5549	1.2192
CL	24	5.14	0.4881	0.5927	4.7082	0.5499	1.1947
SL	24	6.14	0.5153	0.6138	5.4851	0.5760	1.3013
JD	24	5.86	0.5774	0.6173	5.3008	0.5739	1.2757
ZR	24	6.00	0.5357	0.6037	5.3328	0.5659	1.2709
SC	14	4.14	0.5204	0.6750	4.1429	0.5892	1.2059
RZ	24	5.71	0.4702	0.5512	5.0390	0.5127	1.1450
WP	24	6.14	0.5179	0.5966	5.5135	0.5624	1.2889
YP	24	6.43	0.4829	0.6142	5.5779	0.5754	1.3086
PH	24	5.57	0.4622	0.5557	5.0395	0.5216	1.1833
MC	24	6.00	0.5321	0.5910	5.3389	0.5532	1.2486
BX	24	5.00	0.5060	0.6040	4.6105	0.5619	1.2190
ZB	15	5.86	0.4952	0.6230	5.7783	0.5784	1.3145
KX	24	5.57	0.4428	0.5556	4.8706	0.5133	1.1339
LG	24	5.86	0.4286	0.5716	5.0947	0.5322	1.1974
Mean	22.6310	5.73	0.4793	0.5970	5.1439	0.5543	1.2342

*n*, sample size; *N*_A_, number of alleles; *H*_O_, observed heterogeneity, *H*_E_, expected heterogeneity; *A*_R_, allelic richness; PIC, polymorphism information content; *I*, Shannon’s index.

**Table 3:** AMOVA analysis for genetic variation of 24 populations of *S. litura*.

Source of Variation	*d.f.*	Sum of Squares	Variance Components	Variation	Fixation Index	*p*-Value
Among populations	23	101.154	0.04114	1.92%	*F*_ST_ = 0.0191	<0.001
Among individuals within populations	521	1319.110	0.42924	20.02%	*F*_IS_ = 0.2041	<0.001
Within individuals	545	912.000	1.67339	78.06%	*F*_IT_ = 0.2194	<0.001
Total	1098	2332.264	2.14377			

**Table 4:** Genetic differentiation *F*_ST_ (upper right) and *D*′ (lower left) among 24 populations of *Spodoptera litura*.

	XH	ML	CB	FY	HH	QT	HL	TG	HY	BD	CL	SL	JD	ZR	SC	RZ	WP	YP	PH	MC	BX	ZB	KX	LG
XH		0.024	0.038	0.032	0.058	0.035	0.031	0.047	0.069	0.047	0.046	0.032	0.047	0.030	0.123	0.046	0.060	0.032	0.067	0.059	0.077	0.058	0.081	0.101
ML	0.015		0.034	0.039	0.049	0.036	0.040	0.057	0.097	0.049	0.068	0.042	0.048	0.032	0.131	0.058	0.063	0.039	0.066	0.058	0.084	0.081	0.101	0.121
CB	0.024	0.023		0.033	0.054	0.031	0.030	0.036	0.064	0.019	0.038	0.045	0.053	0.036	0.139	0.041	0.036	0.025	0.034	0.030	0.065	0.065	0.104	0.095
FY	0.023	0.027	0.024		0.077	0.032	0.064	0.040	0.087	0.057	0.048	0.043	0.048	0.033	0.139	0.033	0.039	0.042	0.055	0.068	0.101	0.067	0.103	0.095
HH	0.036	0.032	0.035	0.052		0.068	0.051	0.054	0.091	0.058	0.057	0.066	0.055	0.054	0.141	0.066	0.096	0.073	0.050	0.061	0.092	0.097	0.115	0.164
QT	0.027	0.027	0.023	0.024	0.049		0.049	0.054	0.101	0.043	0.059	0.049	0.053	0.025	0.143	0.060	0.036	0.039	0.054	0.079	0.098	0.076	0.097	0.110
HL	0.019	0.025	0.020	0.043	0.032	0.036		0.059	0.067	0.022	0.046	0.041	0.066	0.042	0.114	0.059	0.067	0.029	0.069	0.042	0.086	0.074	0.100	0.120
TG	0.031	0.038	0.025	0.029	0.037	0.040	0.039		0.085	0.041	0.050	0.048	0.062	0.049	0.171	0.051	0.079	0.074	0.035	0.067	0.089	0.047	0.107	0.137
HY	0.048	0.067	0.046	0.064	0.064	0.075	0.048	0.062		0.090	0.048	0.058	0.080	0.083	0.187	0.065	0.101	0.065	0.081	0.085	0.092	0.083	0.128	0.111
BD	0.030	0.032	0.013	0.039	0.038	0.032	0.014	0.029	0.063		0.054	0.050	0.058	0.050	0.149	0.062	0.065	0.034	0.041	0.038	0.080	0.059	0.112	0.130
CL	0.030	0.044	0.026	0.034	0.038	0.043	0.030	0.035	0.036	0.036		0.046	0.060	0.046	0.104	0.054	0.064	0.055	0.065	0.052	0.095	0.073	0.106	0.099
SL	0.020	0.026	0.029	0.030	0.041	0.036	0.026	0.032	0.042	0.032	0.030		0.036	0.034	0.157	0.043	0.070	0.040	0.062	0.069	0.065	0.029	0.063	0.099
JD	0.028	0.030	0.034	0.033	0.035	0.038	0.040	0.041	0.056	0.037	0.039	0.023		0.047	0.131	0.064	0.088	0.046	0.055	0.073	0.080	0.045	0.103	0.123
ZR	0.019	0.021	0.024	0.023	0.035	0.020	0.027	0.034	0.059	0.033	0.031	0.022	0.030		0.141	0.041	0.031	0.036	0.054	0.062	0.081	0.059	0.065	0.085
SC	0.061	0.070	0.075	0.080	0.076	0.086	0.060	0.093	0.108	0.080	0.060	0.081	0.069	0.076		0.166	0.166	0.131	0.192	0.146	0.207	0.202	0.238	0.237
RZ	0.034	0.042	0.031	0.026	0.048	0.046	0.043	0.039	0.051	0.045	0.040	0.032	0.046	0.031	0.099		0.065	0.042	0.067	0.070	0.084	0.069	0.076	0.100
WP	0.037	0.041	0.024	0.028	0.061	0.027	0.042	0.053	0.070	0.043	0.042	0.045	0.054	0.021	0.088	0.048		0.050	0.071	0.072	0.103	0.099	0.105	0.085
YP	0.019	0.025	0.017	0.029	0.046	0.029	0.018	0.048	0.047	0.023	0.036	0.025	0.029	0.023	0.070	0.032	0.033		0.058	0.048	0.060	0.066	0.083	0.075
PH	0.047	0.047	0.026	0.042	0.037	0.042	0.049	0.027	0.062	0.031	0.047	0.044	0.040	0.039	0.109	0.052	0.051	0.042		0.053	0.063	0.062	0.104	0.109
MC	0.038	0.038	0.021	0.047	0.040	0.056	0.028	0.045	0.061	0.026	0.035	0.045	0.047	0.041	0.081	0.051	0.048	0.032	0.039		0.084	0.098	0.135	0.127
BX	0.047	0.053	0.043	0.067	0.058	0.068	0.054	0.059	0.065	0.052	0.062	0.042	0.050	0.052	0.106	0.060	0.066	0.039	0.046	0.055		0.058	0.070	0.085
ZB	0.034	0.049	0.041	0.045	0.059	0.053	0.045	0.032	0.058	0.038	0.046	0.019	0.028	0.038	0.099	0.050	0.061	0.041	0.044	0.061	0.038		0.069	0.090
KX	0.056	0.069	0.072	0.074	0.078	0.072	0.068	0.075	0.093	0.077	0.074	0.046	0.070	0.048	0.130	0.059	0.073	0.059	0.077	0.091	0.051	0.050		0.066
LG	0.063	0.077	0.063	0.066	0.101	0.078	0.076	0.090	0.080	0.084	0.067	0.065	0.078	0.057	0.124	0.072	0.058	0.050	0.078	0.083	0.058	0.059	0.050	

**Table 5:** Correlation coefficient based on the geographic distance of 24 *S. litura* populations.

Distance Class (Km)	N	r
44	56	−0.0380
89	68	0.0261
133	40	−0.0241
178	44	−0.1003
222	38	−0.0584
267	34	−0.0184
311	2	−0.1166
356	2	−0.3507
400	32	−0.0619
448	24	−0.1292
489	60	−0.0679
534	78	−0.0621
578	38	−0.3657
622	32	0.0232

**Table 6:** Emigration rate (upper right) and immigration rate (lower left) across *Spodoptera litura* populations.

	XH	ML	CB	FY	HH	QT	HL	TG	HY	BD	CL	SL	JD	ZR	SC	RZ	WP	YP	PH	MC	BX	ZB	KX	LG
XH	**0.6929**	0.0066	0.1098	0.0061	0.0066	0.0066	0.0063	0.0062	0.0065	0.0064	0.0065	0.0066	0.0065	0.0073	0.0063	0.0202	0.0081	0.0068	0.0129	0.0076	0.0074	0.0064	0.0370	0.0065
ML	0.0620	**0.6731**	0.1080	0.0064	0.0064	0.0066	0.0064	0.0062	0.0065	0.0064	0.0066	0.0066	0.0063	0.0066	0.0065	0.0126	0.0066	0.0064	0.0155	0.0066	0.0068	0.0065	0.0118	0.0065
CB	0.0306	0.0079	**0.7806**	0.0069	0.0078	0.0067	0.0076	0.0072	0.0073	0.0070	0.0070	0.0076	0.0076	0.0073	0.0069	0.0160	0.0081	0.0074	0.0134	0.0085	0.0082	0.0070	0.0180	0.0075
FY	0.0242	0.0078	0.1245	**0.6744**	0.0075	0.0078	0.0075	0.0076	0.0078	0.0076	0.0076	0.0078	0.0080	0.0076	0.0076	0.0115	0.0082	0.0077	0.0116	0.0076	0.0078	0.0078	0.0147	0.0077
HH	0.0508	0.0070	0.1024	0.0072	**0.6739**	0.0072	0.0072	0.0071	0.0072	0.0073	0.0072	0.0070	0.0073	0.0073	0.0072	0.0105	0.0076	0.0073	0.0170	0.0073	0.0071	0.0069	0.0161	0.0071
QT	0.0110	0.0086	0.1216	0.0083	0.0084	**0.6750**	0.0081	0.0080	0.0082	0.0082	0.0083	0.0084	0.0082	0.0085	0.0084	0.0125	0.0092	0.0086	0.0158	0.0086	0.0084	0.0083	0.0132	0.0084
HL	0.0301	0.0074	0.1335	0.0074	0.0070	0.0073	**0.6740**	0.0072	0.0070	0.0072	0.0075	0.0072	0.0073	0.0071	0.0073	0.0094	0.0073	0.0071	0.0109	0.0074	0.0072	0.0072	0.0118	0.0072
TG	0.0235	0.0072	0.1309	0.0067	0.0070	0.0069	0.0072	**0.6737**	0.0070	0.0073	0.0071	0.0072	0.0070	0.0072	0.0069	0.0115	0.0074	0.0070	0.0175	0.0069	0.0075	0.0073	0.0152	0.0070
HY	0.0100	0.0088	0.0988	0.0088	0.0088	0.0088	0.0087	0.0086	**0.6754**	0.0088	0.0086	0.0090	0.0088	0.0086	0.0086	0.0170	0.0090	0.0089	0.0144	0.0090	0.0088	0.0088	0.0262	0.0089
BD	0.0233	0.0072	0.1410	0.0073	0.0070	0.0072	0.0071	0.0069	0.0070	**0.6736**	0.0070	0.0070	0.0070	0.0070	0.0074	0.0087	0.0072	0.0071	0.0144	0.0073	0.0074	0.0073	0.0105	0.0072
CL	0.0336	0.0070	0.1329	0.0069	0.0069	0.0068	0.0070	0.0070	0.0069	0.0068	**0.6737**	0.0068	0.0071	0.0069	0.0069	0.0098	0.0070	0.0069	0.0084	0.0071	0.0068	0.0069	0.0171	0.0067
SL	0.0256	0.0068	0.1200	0.0068	0.0070	0.0069	0.0071	0.0069	0.0069	0.0069	0.0070	**0.6737**	0.0071	0.0067	0.0069	0.0098	0.0074	0.0071	0.0096	0.0072	0.0074	0.0070	0.0350	0.0070
JD	0.0445	0.0069	0.1190	0.0072	0.0071	0.0070	0.0071	0.0069	0.0069	0.0071	0.0068	0.0069	**0.6737**	0.0071	0.0069	0.0080	0.0072	0.0071	0.0099	0.0073	0.0072	0.0069	0.0181	0.0071
ZR	0.0277	0.0072	0.1267	0.0068	0.0072	0.0072	0.0073	0.0070	0.0072	0.0071	0.0070	0.0071	0.0071	**0.6738**	0.0069	0.0101	0.0075	0.0072	0.0093	0.0072	0.0071	0.0070	0.0241	0.0071
SC	0.0828	0.0090	0.0484	0.0088	0.0088	0.0092	0.0091	0.0086	0.0089	0.0088	0.0090	0.0088	0.0087	0.0088	**0.6755**	0.0113	0.0087	0.0086	0.0103	0.0088	0.0088	0.0087	0.0125	0.0089
RZ	0.0220	0.0071	0.1280	0.0069	0.0070	0.0068	0.0068	0.0070	0.0071	0.0070	0.0069	0.0071	0.0070	0.0068	0.0069	**0.6816**	0.0075	0.0071	0.0209	0.0072	0.0073	0.0071	0.0139	0.0070
WP	0.0177	0.0069	0.1379	0.0070	0.0069	0.0070	0.0074	0.0072	0.0070	0.0071	0.0070	0.0071	0.0069	0.0071	0.0068	0.0150	**0.6745**	0.0073	0.0094	0.0070	0.0074	0.0070	0.0186	0.0068
YP	0.0294	0.0072	0.1233	0.0070	0.0072	0.0072	0.0072	0.0072	0.0070	0.0073	0.0069	0.0072	0.0072	0.0072	0.0071	0.0109	0.0077	**0.6740**	0.0129	0.0075	0.0080	0.0071	0.0197	0.0070
PH	0.0084	0.0070	0.1388	0.0068	0.0070	0.0070	0.0070	0.0071	0.0071	0.0069	0.0069	0.0069	0.0067	0.0070	0.0070	0.0091	0.0073	0.0067	**0.6854**	0.0072	0.0079	0.0068	0.0249	0.0071
MC	0.0274	0.0066	0.1462	0.0071	0.0070	0.0068	0.0067	0.0072	0.0069	0.0069	0.0068	0.0069	0.0068	0.0070	0.0070	0.0102	0.0073	0.0070	0.0082	**0.6739**	0.0073	0.0067	0.0092	0.0070
BX	0.0301	0.0068	0.0467	0.0071	0.0071	0.0072	0.0071	0.0072	0.0069	0.0070	0.0069	0.0070	0.0069	0.0071	0.0071	0.0105	0.0072	0.0071	0.0085	0.0071	**0.6746**	0.0071	0.1026	0.0069
ZB	0.0179	0.0086	0.0761	0.0087	0.0089	0.0090	0.0087	0.0088	0.0086	0.0089	0.0086	0.0088	0.0090	0.0091	0.0090	0.0125	0.0090	0.0089	0.0123	0.0091	0.0097	**0.6755**	0.0448	0.0089
KX	0.0142	0.0071	0.0239	0.0078	0.0073	0.0073	0.0105	0.0085	0.0070	0.0092	0.0082	0.0080	0.0081	0.0072	0.0078	0.0109	0.0077	0.0082	0.0110	0.0073	0.0084	0.0096	**0.7868**	0.0076
LG	0.0137	0.0068	0.0165	0.0069	0.0068	0.0069	0.0069	0.0069	0.0069	0.0070	0.0068	0.0069	0.0069	0.0068	0.0070	0.0102	0.0070	0.0069	0.0080	0.0071	0.0070	0.0068	0.1536	**0.6737**

## Data Availability

Not applicable.
